# Communication in the Cancer Microenvironment as a Target for Therapeutic Interventions

**DOI:** 10.3390/cancers12051232

**Published:** 2020-05-14

**Authors:** Agnieszka Dominiak, Beata Chełstowska, Wioletta Olejarz, Grażyna Nowicka

**Affiliations:** 1 Department of Biochemistry and Pharmacogenomics, Faculty of Pharmacy, Medical University of Warsaw, 02-097 Warsaw, Poland; wioletta.olejarz@wum.edu.pl (W.O.); grazyna.nowicka@wum.edu.pl (G.N.); 2 Center for Preclinical Research, Medical University of Warsaw, 02-097 Warsaw, Poland; 3 Department of Internal Medicine and Hematology, Laboratory of Hematology and Flow Cytometry, Military Institute of Medicine, 04-140 Warsaw, Poland; beata-chelstowska@wp.pl

**Keywords:** tumor microenvironment, communication in cancer, therapeutic target, oncology therapy

## Abstract

The tumor microenvironment (TME) is a complex system composed of multiple cells, such as non-cancerous fibroblasts, adipocytes, immune and vascular cells, as well as signal molecules and mediators. Tumor cells recruit and reprogram other cells to produce factors that maintain tumor growth. Communication between cancerous and surrounding cells is a two-way process and engages a diverse range of mechanisms that, in consequence, can lead to rapid proliferation, metastasis, and drug resistance, or can serve as a tumors-suppressor, e.g., through tumor–immune cell interaction. Cross-talk within the cancer microenvironment can be direct by cell-to-cell contact via adhesion molecules, electrical coupling, and passage through gap junctions, or indirect through classical paracrine signaling by cytokines, growth factors, and extracellular vesicles. Therapeutic approaches for modulation of cell-cell communication may be a promising strategy to combat tumors. In particular, integrative approaches targeting tumor communication in combination with conventional chemotherapy seem reasonable. Currently, special attention is paid to suppressing the formation of open-ended channels as well as blocking exosome production or ablating their cargos. However, many aspects of cell-to-cell communication have yet to be clarified, and, in particular, more work is needed in regard to mechanisms of bidirectional signal transfer. Finally, it seems that some interactions in TEM can be not only cancer-specific, but also patient-specific, and their recognition would help to predict patient response to therapy.

## 1. Introduction

Despite many efforts, cancer is one of the main causes of human deaths. According to the World Health Organization, it was responsible for approximately 9.6 million deaths in 2018. It is generally accepted that the fight against cancer must be multidirectional and involve the development of new strategies for preventive action, early diagnosis, and treatment to enhance effectiveness and precision of cancer therapy, increase patients survivability, and improve their quality of life [1,2,3]. However, current standards therapy often overlooked the assumption that cancer is an “ensemble production”. Apart from malignant cells, there are lots of supporting players, including fibroblasts, pericytes, endothelial cells, adipocytes, bone-marrow-derived mesenchymal stem cells, and immune cells. Each of these stromal cell types plays a role in tumor proliferation, metastasis, and treatment failure [4,5]. The extracellular matrix (ECM) is a highly dynamic structure that surrounds the above-mentioned cells and affects their proliferation and cell–cell communication via the transmission of mechanical signals and cell adhesion [6]. ECM constituents mainly derive from the tumor cells themselves but also, to a large degree, from cancer-associated fibroblasts (CAF). High amounts of metalloproteinases in the cancer niche process ECM components and are involved in ECM remodeling, resulting in the release of various signaling molecules with both pro- and anti-tumor activities [7].

Cell communication is required for proper cellular activities or movements, and both failure and excess of this cross-talk can lead to tissue pathology. Normal and cancerous cells dynamically transmit reciprocal information, and, by contacting the tumor stromal cells, acquire a pro-tumoral phenotype that can promote cancer progression. Cells in this microenvironment are also involved in tumor suppression, and, for example, the accumulation of cytotoxic CD8+T cells and Th1 cells in tumor stroma suggests that the immune system fights against cancer. However, some immune cells, such as tumor-associated macrophages, can promote cancer development, indicating that immune cells have a multifaceted role [8]. Thus, increasing attention is being paid to fully understand the mechanism of interaction between cancer and the surrounding cells.

Currently, many studies have documented that the vital role in tumor progression plays on a complex system of intercellular communication via direct cell-to-cell contact or through classical paracrine/endocrine signaling. The most common type of signal transition to neighboring or long-distance cells is the secretion of soluble factors into the extracellular space, like cytokines, chemokines, and growth factors. Another way of cell interaction is through adhesion molecules and gap junctions. Recent research has also highlighted that non-cancer cells can donate healthy mitochondria and other organelles by tunnel nanotubes to keep cancer cells alive, but it was also reported that horizontal mitochondrial transfer is possible from cancer cells to surrounding cells (e.g., from cancer to stromal cells) [9,10,11]. An important way of cells to cross-talk is membrane vesicle secretion that does not need specific receptors to reach target cells. Moreover, cancerous cells create a hypoxic and acidic microenvironment. Reduction of the pH (ranging between 6.0 and 6.5) can impact surrounding cells and repress their antitumor activity [12]. Hypoxia can support cancer growth through the differentiation of fibroblasts into CAFs [13]. However, the main mechanism of fibroblast activation is a cross-talk involving Notch and JAK1/STAT3 signaling pathways, and another way is by a range of inflammatory signaling molecules, for instance, IL-1 acting via NF-κB and IL-6 acting through signal STAT transcription factors. Similarly, transforming growth factor β (TGFβ) family ligands and lysophosphatidic acid are also involved in activating signals for fibroblasts, while cytokine, a leukemia inhibitory factor (LIF), is known as a sustainer of their invasive phenotype [14,15,16,17].

Understanding the mechanism of cell–cell communication and cross-talk between a tumor and its microenvironment is of great importance for the development of effective cancer treatments. Thus, in this review, we present a comprehensive, up-to-date overview of communication phenomena in cancer and show newly proposed therapeutic methods affecting cell cross-talk.

## 2. Mechanisms of Cellular Communication

**Figure 1:**
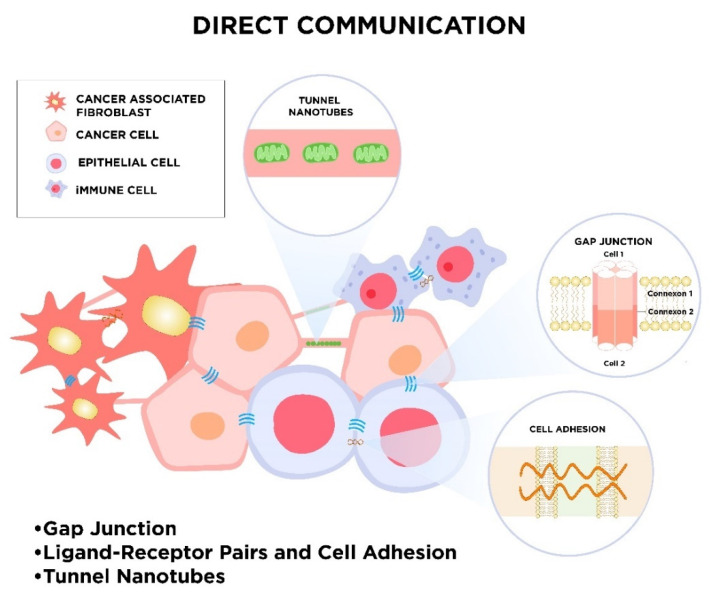
The schematic visualization of direct communication in the tumor microenvironment. The sharing of information between cancer and stromal cells, such as non-cancerous epithelial cells, cancer-associated fibroblasts, immune cells, and others, can occur in different ways, e.g., via gap junction, open-ended cytoplasmic channels (tunnel nanotubes), or through direct ligand–receptor signaling and cell adhesion [18]. These dynamic interplays effectively enable the exchange of cellular cargos over short or long (tunnel nanotubes) distances. For instance, via tunnel nanotubes, mitochondria can be donated from one cell to another in two directions. Gap junctions joint the same and different types of cells and consist of connexions that include six connexins. They are able to transfer rather small molecules and metabolites [19]. Similarly, physical cell–cell attachments via adhesion proteins are impermeable for macromolecules.

**Figure 2:**
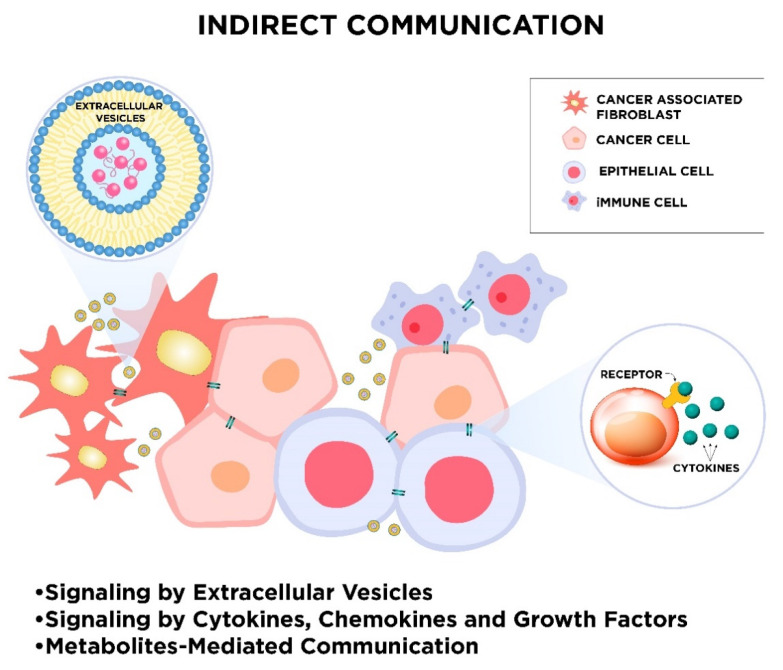
The schematic visualization of indirect communication in the tumor microenvironment. The sharing of information between cancer and stromal cells, such as non-cancerous epithelial cells, cancer-associated fibroblasts, immune cells, and others, can occur in different ways, e.g., via extracellular vesicles or through signaling by cytokines, chemokines, and growth factors as well as metabolites-mediated communication. Exosomes can transfer many bioactive molecules over long and very long distances. Secreted signaling molecules can also travel to remote cells.

During evolution, many modes of cell-to-cell communication have developed. These appearing forms of intercellular communication turn out to be essential for the maintenance of physiological cell functions and cellular response to external stimuli. Due to the fundamental role of cell cross-talk, different cell-to-cell signaling mechanisms parallelly exist. One way of cell signaling is direct intercellular communication, which includes direct cell-to-cell contact by electrical coupling, direct passage through gap junctions, direct receptor–ligand binding of membrane proteins, or a mechanism that relies on nanotubular cell-to-cell connections termed “membrane nanotubes” (MNTs; Figure 1). Cells also communicate with each other indirectly via signal molecules and mediators, as well as transferring biological information by extracellular vesicles (Figure 2).

### 2.1. Direct Intercellular Communication

#### 2.1.1. Gap Junctions

Gap junctions (GJs) are major contributors to the maintenance of cell homeostasis. When the cells are a short distance apart, they can be connected with each other using gap junctions, which allow various molecules, ions, and electrical impulses to pass directly between cells. The gap junction channel is composed of two connexons (hemichannels), which connect cells across the intercellular space. The connexons are formed by hexameric oligomers of transmembrane proteins, the connexins (Cxs) [20]. Approximately 21 Cxs are identified in humans. Most cell types express multiple connexin isoforms, which allow heteromeric hemichannels and heterotypic gap junctions to form, which could possibly provide a structural basis for the selectivity of GJs and may have overlapping or distinct functions [21]. The Cx43, being more widespread than other Cxs, is expressed mainly in a few tissues only. The regulation of GJs can be modulated by connexin-associated proteins, including regulatory phosphatases, cytoskeletal elements, and enzymes. Key regulatory pathways act through phosphorylation/dephosphorylation of Cxs, mainly at the C-terminal domain. Phosphorylation determines disassembly and internalization of the GJ, due to the disengagement of Cxs from the GJ, whereas unphosphorylated proteins remain in GJ [22]. Already in the 1970s, scientists have observed a loss of functional GJs in cancer cells and considered that GJs were involved in carcinogenesis [23]. A number of biological and chemical substances (also suspected to participate in cancerogenesis), including toxins, organic solvents, pesticides, pharmaceuticals, peroxides, metals, and phthalates, are able to inhibit GJs [24].

A number of studies have highlighted the role of Cxs and GJs in carcinogenesis and their involvement in numerous diseases. The loss of GJ or Cxs is associated with abnormal cell proliferation, and the deregulation of GJs is observed in cancer [25,26,27]. Additionally, Cxs themselves can promote tumor cell growth and invasiveness, contributing to the overall tumorigenicity [28]. Some Cxs (as Cx26, Cx37, and Cx43) may be crucial in metastasis, and intact GJs may act as tumor suppressors in the initial stages of tumorigenesis, controlling cell proliferation. Depending on the state and function of a cell, inhibition of GJs may lead to uncontrolled proliferation (promotion). Re-expression of Cxs in migrating tumor cells was found to promote tumor metastasis [29]. It was also recognized that Cxs and GJs can protect cells from cancer [21]. Therefore, Cxs can act as both inhibitors of cell proliferation and inducers of cell migration, invasion, and activation of cancer cell migration out of the tumor core [30,31].

#### 2.1.2. Ligand–Receptor Pairs and Cell Adhesion

Adjacent cells can communicate through contact-dependent signaling. Physical contact of one cell with a recipient cell is required. Receptors and ligands can occur as membrane-anchored molecules, and their interaction results in effective membrane adhesion. The ligand binds to membrane receptors expressed by other cells, initiating a specific signaling pathway and response in the target cell, but ligands rather do not diffuse from the producing cell [32,33]. Adhesion molecules take part in juxtacrine stimulation and are receptors that consist of an intercellular domain that interacts with cytoplasmic proteins and an extracellular domain that binds to ligands [34,35]. Adhesion molecules are commonly divided into integrins, selectins, cadherins, and the members of the immunoglobulin superfamily. Different adhesion molecules bind to different ligands. Cadherins, selectins, and immunoglobulin superfamily members are involved in cell–cell adhesion. Integrins typically joint the cell with its extracellular matrix, but immune cell integrins also bind to soluble ligands and ligands on other cells. Many proteins of ECM, in particular laminins, fibronectin, collagens, and vitronectin, are ligands for integrins. Due to their function, adhesion molecules are part of an adhesive network. Their expression not only changes in cancer cells but also in non-cancerous cells of TME [36].

Moreover, the epithelial barriers for cancer cell migration are made by intercellular adhesion complexes, including tight junctions (TJs), adherens junctions (AJs), and desmosomes. These connections are mainly characteristic of epithelial and endothelial cells. Networks called “tight junctions” are formed in areas where the cell membranes, joined by transmembrane proteins and cell cytoskeletons, are fused. These connections mediate ion or other molecules transport and osmosis as well as dictate cell polarity [37,38]. Up- or downregulation of TJ proteins in cancers results in the loss of cell–cell association, thus can lead to uncontrolled growth and metastasis [39]. A correlation between the loss of TJs and tumor differentiation has been observed [40]. AJs are mainly composed of cadherins on adjacent cells, cadherin adhesion receptors, and the catenin protein family [41]. Downregulation of E-cadherin was found to be associated with progression, promotion, and poor prognosis, and changes in E-cadherin expression were documented in gastric, prostate, liver, and colon cancers [42,43,44]. In addition, in prostate, gastric, colorectal, cervical, bladder, breast, skin, and endometrial cancer, modifications of the expression of desmosomal elements have also been observed [45,46].

#### 2.1.3. Tunnel Nanotubes called “Intercellular Bridges”

Long-distance cell-to-cell communication is possible by the formation of tunneling nanotubes (TNTs) and tumor microtubes (TMs), which allow cells to exchange different chemical and biological material between cells and also facilitate electrical long-range coupling. TNTs are long and thin (50–200 nm) extensions of the cell cytoplasm formed by F-actin filaments that form open-ended channels [47]. They occur in many types of cells, e.g., immune cells (like macrophages), two types of lymphocytes (natural killers and B-cells), and also in dendritic cells. It has been reported that TNTs are formed between endothelial progenitor cells and endothelial cells, renal proximal tubular epithelial cells (RPTEC), retinal pigment epithelial cells, and also between cardiac myocytes [48,49]. TNTs are also extensively used for cancerous cell communication. Formation of those structures has been observed in vitro in cell lines of colon carcinoma cells, MCF7 and MDA-MB-231 breast cancers, and also in bladder cancer cells [10,50,51]. Described nanotubes either connect cancer cells together or cancer cells with normal stromal cells (e.g., B-cell precursor acute lymphoblastic leukemia cells and acute myeloid leukemia cells) [52].

TNTs have been recognized as a novel mode of intercellular communication, enabling the transfer of mitochondria, cellular vesicles, miRNAs, viral particles, and some proteins. In cancer cells, TNT formation may correlate with invasiveness. Moreover, factors that can be transferred by TNTs have been shown to promote chemoresistance [53,54].

Little is known about TNT biogenesis, but it has been recognized that TNTs contain an array of cytoskeletal filaments of which F-actin is the most abundant. The presence of calcium-sensitive dynamin-related Rho-GTPases (Miro1 and Miro2), KLF 5 kinesin motor protein, and accessory proteins like TRAK 1 and TRAK2 were reported in the structure of TNTs. Myo 19 and Myo 10 permit the efficient shipping of cargo between cells via an actin–myosin-dependent mechanism. The transported cargo includes large organelles like mitochondria and lysosomes [55,56]. Different conditions may promote TNT formation. Wang et al. demonstrated a significant contribution of p53, EGFR, Akt, PI3K, and also mTOR activation in neurons and astrocytes. They also recognized that cells under stress conditions always develop TNTs with unstressed cells [57]. In this experimental study, hydrogen peroxide, serum depletion, and some cytokines are known as factors that favor TNT formation. These structures, particularly under stress or injury conditions, transfer mitochondria and other cellular components between numerous cell types, including endothelial, epithelial, cardiac, renal, and immune cells [58]. TMs are larger and longer-lived (active from several days to a month) than TNTs (active for minutes). TMs are composed of Cx43 gap junction protein and were recognized to promote aggressiveness, invasion, and therapeutic resistance of brain cancer cells [59,60].

Both TNTs and TMs, and GJs, allow a direct exchange of cytoplasmic molecules between connected cells, therefore, they participate in key biological processes, including development, immune response, and signaling, and are also involved in the pathogenesis of several diseases, including HIV and neurodegenerative and cancerous diseases [61]. However, there are some differences between TNTs (TMs) and GJs. The distance required to establish cellular membrane contact must be short in forming GJs, while TNTs allow long-distance communication. Secondly, these structures transport molecules of varying sizes. GJs are able to transport only small molecules (up to 1.2 kDa), including second messengers and small peptides, whereas TNTs are able to transfer both small molecules as well as mediate the exchange of large organelles and vesicles. Although both TNTs and GJs are known to mediate cell-to-cell interactions, still little is known whether and how these two direct intercellular communication systems interact with each other [62].

### 2.2. Indirect Intercellular Communication

#### 2.2.1. Signaling by Extracellular Vesicles

Normal and cancerous cells secrete small membrane-bound vesicles in cup-shaped called extracellular vesicles (EVs), which circulate in body fluids and are finally taken up by proximal or distal recipient cells. However, EVs are characterized by a short half-life; a few minutes after infusion, up to 90% of them are eliminated from the bloodstream [63]. Based on particle diameter and mechanism of formation, three types of EVs have been identified: exosomes (with 40–120 nm diameter), microvesicles (MVs, with 50–1000 nm diameter), and apoptotic bodies (Abs, with 500–2000 nm diameter) [64,65]. EVs carry packages of information located inside or on their surface. Their cargo composition depends on the cell-type origin and varies between cell conditions. As a result of fusion, receptor-mediated internalization or endocytosis of the EVs with the plasmalemma of the recipient cell, transported proteins (enzymes, membrane proteins, heat shock proteins, transcription factors), mRNA, tRNAs, non-coding RNAs (LncRNAs), microRNAs and DNA, and lipids (such as sphingolipids, ceramides, cholesterol, and saturated fatty acids) are released, and affect the recipient cell’s metabolism and can induce cell transformation [66,67].

Available data indicate that different factors can modify EV release, and so, for example, in response to intracellular Ca^2+^ accumulation or microenvironment acidification, their secretion is increased [68,69]. In the blood of cancer patients, twice more exosomes were found than in the blood of healthy individuals [70,71]. A high amount of cancer-derived exosomes is associated with poor prognosis [72].

Exosome-mediated interaction between cancer and stromal cells leading to the transfer of tumor-derived bioactive molecules can modify the TME. Tumor-derived EVs, called TEVs, play particular roles in cancer initiation, progression, and metastasis, as shown in ovarian [73], colorectal [74], breast [75], melanoma [76], and prostate cancer [77]. Moreover, TEVs are suppliers of multidrug resistance-associated proteins involved in treatment failure development. Corcoran and colleagues showed that in prostate cancer, EVs convey docetaxel resistance [78]. This phenomenon was also observed in lung, breast, and liver cancers [79,80,81]. Simultaneously, communication via exosomes is bidirectional; EVs can be donated from cancer cells, but can also reach malignant cells. Exosomes are secreted by different cells as intestinal epithelial cells, platelets, mastocytes, antigen-presenting cells, fibroblasts, hepatocytes, and lymphocytes, and EVs of different origins can interact with cancer cells [82]. Exosomes derived from CAF can provide nutrients to malignant cells and support tumor growth [83]. Apart from the well-known effects of exosomes on the differentiation of surrounded cells, TEVs can also mediate tumor-stem/progenitor cell communication to create pro-tumorigenic microenvironments [84,85]. It was observed that through transfer of the oncoprotein MET, melanoma-derived exosomes stimulate vasculogenic and hematopoietic bone marrow progenitor cells [86]. Tumor-derived exosomes are also involved in tumor immune escape as extracellular vesicles expressing FasL secreted from melanoma cells induce Fas-mediated apoptosis in T-cells. However, it cannot be ruled out that at early stage of cancer, exosomes have antitumor functions [87].

#### 2.2.2. Signaling by Cytokines, Chemokines, and Growth Factors

Various cytokines, chemokines, and growth factors are involved in cell–cell cross-talk within tumor and cancer environments, as well as in communication between cancerous and non-cancerous cells [88]. Cytokines and chemokines are key players in the development of cancer-related inflammation, with consequent direct and indirect effects on tumor cells [89]. Cytokines are secreted in response to different cellular, developmental, and/or environmental stresses and act via cytokine receptors on target cells, affecting certain intracellular signaling pathways to promote a specific cellular response. They affect not only the above-mentioned closely adjacent cells, as well as acting at a distance, and may also affect the cell of their origin. Cytokines can both inhibit cancer development and progression as well as promote its growth and enhance invasion. Interleukins are produced not only by immune cells but also by cancer cells, and cancer cells express interleukin receptors. There is strong evidence that interleukins such as IL-1, IL-4, and IL-6, as well as IL-8 and IL-10, promote tumor development [90,91,92,93,94]. IL-6 levels are correlated with cancer progression and inversely correlated with patient response to treatment and survival [95]. Other interleukins as IL-2 and other members of this family, such as IL-15 and IL-21, as well as IL-12, have been recognized to activate host immune systems against cancer [96,97,98].

Tumor necrosis factor α (TNFα) and TGFβ signaling affect the tumor environment, tumor progression, and drug resistance [99]. Current data underline a complex impact of these factors on tumorigenesis and cancer therapy [100,101,102]. Tumor necrosis factor-related apoptosis-inducing ligand (TRAIL) is able to selectively induce apoptosis in cancer cells [103]. Moreover, interferon-gamma (IFN-gamma) can protect against tumor development, affecting both innate and adaptive host immune responses [104].

Chemokines, a large family of cytokines with chemotactic activity, mediate leukocyte migration and are relevant for cancer-related inflammation. The majority of cancer cells produce chemokines, as well as their receptors. In general, chemokine expression at the tumor site promotes leukocyte infiltration and anti-tumor immune responses [105], while the expression of chemokine receptors by cancer cells promotes their growth and metastasis. Therefore, tumor-derived chemokines can both inhibit and stimulate tumor growth. Monocyte chemoattractant protein 1 (MCP-1, also known as CCL2) and other chemokines as CXCL1, CXCL3, CXCL5, and CCL8 can act as mediators of angiogenesis and tumor progression [106], while those without the ELR motif (CXCL4, CXCL9, CXCL10, CXCL11, and CXCL14) have been shown to inhibit angiogenesis [107]. Additionally, F-box proteins, which are subunit recruiting modules of SCF (SKP1-Cullin 1-F-box protein) E3 ligase complexes, influence cell proliferation, invasion, and metastasis, therefore, are involved in cancer development and progression [108].

Vascular endothelial growth factor (VEGF) and their receptors—VEGFR-1, VEGFR-2, VEGFR-3, neuropilin (NRP)-1, and NRP-2—affect tumor angiogenesis by upregulation of expression of a variety of growth factors [109]. EGF, a member of a family of peptide growth factors and the EGF/EGF receptor signaling pathway, is involved in cell proliferation, differentiation, and migration [110]. Expressions of the EGF and its receptor (EGFR) are correlated with tumor growth and metastasis [111]. Additionally, placental growth factor (PlGF) is an important regulator involved in controlling angiogenic and inflammatory responses by the formation of PlGF/VEGF homodimer/heterodimer and VEGF-competitive binding to the VEGF receptors and sFlt-1 [112]. Furthermore, the enhanced activity of platelet-derived growth factor (PDGF) receptor signaling may promote tumor development [113]. Moreover, dysregulation of the IGF-axis (insulin-like growth factor) is involved in the oncogenesis and metastasis of various solid tumors [114].

Nowadays, modern tools, including transcriptome analyses, are used to map ligand and receptor protein expression. They also identify cell types and ligand–receptor interactions, as well as cross-talk between TME cells and TME and cancer cells [115,116,117]. Expression of some ligands and receptors has been recognized to be restricted to the specific cell types, while some are broadly expressed. Recently, based on bulk RNA sequencing and single-cell RNA sequencing data of more than six thousand glioma cells, the intercellular communication between cancer stem-like cells and macrophages via ligand–receptor interactions have been identified [118]. Ligand–receptor pairs related to invasion and angiogenesis have been recognized, as well as ligand–receptor pairs associated with clinical outcomes and patient survival risks.

#### 2.2.3. Metabolites-Mediated Communication

Cancer cells during development alter their biochemical pathways towards increased glucose usage and promote its fermentation to lactate regardless of the amount of oxygen. The accumulation of lactate leads to acidification of the microenvironment. It is hypothesized that lactate is a stimulator of M2 tissue-associated macrophage polarization [119] and also activates the secretion of VEGF, TGF-β, as well as hypoxia-inducible factors (HIF-1). Moreover, cancer cells via intensified glycolysis limit glucose availability for tumor-infiltrating lymphocytes. In turn, the lack of glucose reduces the effector functions of immune cells [120]. To meet the high energy requirements, cancer cells need other fuel sources. It was documented that exosomes derived from CAF deliver metabolite cargo to prostate cancer cells. Among them, there are acetate, lactate, and TCA cycle metabolites: citrate, pyruvate and fumarate [83,121]. Tumor cells use these energy-rich metabolites in the TCA cycle to produce ATP. Highly glycolytic CAFs can donate their mitochondria to neighboring cancer cells, enhance their respiration, and facilitate tumor growth [122]. Additionally, CAF-derived cytokines like CCL5, IL6, and CXCL10 can promote the TCA cycle and thus favor the proliferation of cancer cells [123]. In addition to changes in glucose metabolism, cancer cells are characterized by changing the rates of fatty acid synthesis.

Bioactive lipids and fatty acids are mediators in the cross-talk between cancer cells and stroma [124]. Fatty acids and other lipids can be transported from stroma cells, especially from adipocytes present in the cancer microenvironment, to cancer cells by lipoproteins or exosomes. Fatty acids taken up by cancer cells and transported by fatty acid-binding proteins (FABPs) are used for energy production for rapid tumor growth and synthesis of prostaglandins, known as potent signaling molecules, and other lipid-derived molecules, which may contribute to cancer development [125] Upregulation of FABP4 in omental metastases compared to the primary ovarian tumor was reported [126]. High FABP3 and FABP4 expressions observed in non-small cell lung cancer were associated with tumor metastasis and negatively affect patient survival [127]. Prostaglandins promote tumor growth in the paracrine mode and coordinate complex interactions between tumor cells and surrounding stromal cells [128]. Cyclooxygenase 2-derived prostaglandin E2 is a potent inducer of the angiogenic switch during mammary cancer progression [129]. PGE2 induces increased proliferation, migration, and invasiveness of colorectal carcinoma cells by phosphorylation of epidermal growth factor receptor EGFR and activation of the PI3K/AKT/mTOR signaling pathway [130]. It was shown that prostaglandin E2 (PGE2) induced the activation of suppressor cells of myeloid origin, which in turn promoted tumor progression of breast cancer [131]. Furthermore, PGE2 promoted the differentiation of monocytes into tumor-associated suppressing macrophages in cervical cancer [132]. Tumor-derived PGE2 can also activate CAF to enhance the secretion of kynurenine, a tryptophan metabolite, which, in turn, increases cancer cell invasiveness [133]. Leukocyte-derived leukotrienes were reported to selectively expand the group of breast cancer cells with high tumorigenic potential and support metastasis [134].

Enhanced levels of lysophosphatidic acid, found in the blood and ascites of ovarian cancer patients, can promote the formation of inflammatory cytokines, which favor the survival of malignant cells and predetermine their more aggressive behavior [135]. Sphingosine-1-phosphate (S1P), an important bioactive sphingolipid, is involved in angiogenesis and lymphangiogenesis, facilitating tumor growth and metastasis [136]. High levels of extracellular S1P induced by enhanced expression of a regulatory sphingosine kinase increase migration and efficiency of vessel formation upon combined cultivation of tumor cells and lymphatic endothelial cells [137].

## 3. New Therapeutic Perspectives Targeted at Cellular Communication

### 3.1. Gap Junction

Connexins, as indicated before, are involved in gap junctions. Only a few of these human family members have been widely studied and characterized in terms of their role of channel-forming to cancer communication. Cx43 has the greatest capacity to transport molecules than the other members and therefore is the object of interest for the oncological field [138]. It was indicated that modification of Cx43 expression in cancer cells can result in a change in sensitivity to chemotherapeutic agents. Previous reports showed that the cancer phenotype can be related to the decline of coupling. However, recent studies in clinical samples revealed that connexins are correlated with metastatic lesions in cancer patients [139,140,141]. Therefore, up- or downregulation of the expression of the connexin-encoding gene should depend on the type as well as the stage of disease development. Hence, two therapeutic options are being considered: the first attempts to focus on the attenuation of Cx43 function and the second is based on chemical agents that enhance its activity. Jensen et al. showed that treatment with carbenoxolone, a Cx43 inhibitor, leads to the death of thyroid cancer cells in spheroids without affecting adherent thyroid cancer cells [142]. On the other hand, it has been documented that the loss of gap between cells resulted in a decline of cell–cell connections and favored invasion and metastatic. Hence, restoring gap junction is one of the therapeutic options that enable the diffusion of drugs and effective reduction of cancer growth. Kong et al. demonstrated that increased Cx43 expression in breast cell line MCF-7, although all-trans retinoic acid (ATRA) treatment resulted in enhanced drug permeability and higher cell chemosensitivity. Moreover, ATRA, given in combination with suicide gene therapy, led to a six-fold increase in apoptosis of MCF-7 cells compared to controls [143]. Second-generation analogs of quinolines (PQs) are a new class of drugs that increase the expression of Cx43 in human breast cancer cells. Heiniger et at. demonstrated that after seven injections of PQ7 in nude mice with T47D xenografts, 100% decrease of tumor growth was observed compared to controls [144]. The latest study discovered a novel role of Cx43-based gap junctions in breast cancer bone metastasis that promote calcium flow from osteogenic cells to the cancer. The combination of carbenoxolone and arsenite trioxide, gap junction inhibitors, blocked such calcium transfer and reduced progression of bone metastasis in mice [145]. Due to available evidence that Cxs may have a diverse role in cancer cell dissemination, the therapeutic interventions aimed at Cx43 seem to be complicated. Despite that, currently, a clinical trial is underway for testing meclofenamate, the modulator of gap junctions, in patients with brain metastasis (ClinicalTrials.gov Identifier: NCT02429570).

### 3.2. Ligand–Receptor Pairs and Cell Adhesion

Following the fact that direct ligand–receptor interactions and cell adhesion can contribute to the progression of cancer, contact-dependent signaling has been extensively studied, and now, anticancer therapy that targets juxtacrine signaling can be used. PD-L1, overexpressed on cancer and non-cancer cells, binds to PD-1 receptors that are located on the activated T-cells. This connection entirely halts or limits the response of the cytotoxic T-cells [146]. Hence, several monoclonal antibodies blockading the PD-1/PD-L1 interaction and achieving encouraging clinical outcomes are available. Nivolumab and pembrolizumab are drugs that belong to this group and are used in the treatment of patients with metastatic melanoma and lung cancers (ClinicalTrials.gov Identifier: NCT02066636; ClinicalTrials.gov Identifier: NCT02180061) [147,148,149,150]. Moreover, currently, a few integrin antagonists are under investigation. Cilengitide, a cyclic RGD pentapeptide, is an anti-angiogenic candidate that targets the integrins αvβ3, αvβ5, and α5β1 and now its efficacy and safety are assessed in the newly diagnosed glioblastoma patients (ClinicalTrials.gov Identifier: NCT00689221). Similarly, the monoclonal antibody specifically binding to α5β1 integrin volociximab is under clinical study in patients with metastatic pancreatic cancer (ClinicalTrials.gov Identifier: NCT00401570) and in advanced non-small-cell lung cancer [151]. Furthermore, cadherins are also potential targets in cancer treatment. ADH-1 that blocks N-cadherin is being assessed in subjects with solid tumors (ClinicalTrials.gov Identifier: NCT00225550).

### 3.3. Tunnel Nanotubes

TNTs are protrusions in actin-containing membranes that are formed in excess in many pathological conditions; for instance, malignant pleural mesothelioma is rich in TNTs. The application of dielectrophoretic force inhibited TNTs formation in mesothelioma by 60% over 72 h and made these cells sensitized to the cytotoxic effect of conventional chemotherapy [152,153]. Other studies have confirmed that TNTs are upregulated in many invasive cancer cells, and their further formation is a response to hypoxic conditions or chemotherapeutic drugs. Doxorubicin, in a dose-dependent manner, stimulated TNT formation in pancreatic cancer cells [154]. Both metformin, an antidiabetic drug, and everolimus, an mTOR inhibitor, suppressed TNT formation in ovarian tumor-derived epithelial and ovarian adenocarcinoma cells [155].

In the brain cancers, mainly glioblastoma (GB) TMs are with high probability responsible for primary and adaptive resistance to all standard treatment options, and pharmacological inhibition of those structures may be a new approach to lower resistance and recurrence rate in GBs [156].

### 3.4. Extracellular Vesicles

Exosomes are recognized as critical mediators in cell communication both locally and distantly, thus contributing to the development of cancer. Many studies have reported that exosomes secreted from malignant cells exert pro-cancerogenic effects [157,158]. Moreover, intercellular transfer of exosomal miRNAs into cells can lead to modification of gene expression and result in drug resistance. Therefore, exosomes have become a new therapeutic target. Such potential strategies focus mainly on blocking exosome production, reduction of their uptake, or ablating exosomal cargos. Interfering with syndecan-syntenin-ALIX signaling regulates mechanism-mediated exosome biogenesis and has detrimental effects on their secretion. It was showed that Rab27 promotes exosome secretion in many types of cancer, and its knockdown significantly attenuates cancer proliferation. High tumor expression of Rab27A or Rab27B is associated with poor survival of patients with hepatocellular carcinoma [159]. Further, the increase of exosome release from CAFs was reported to be a result of their exposition to gemcitabine. In turn, an increased amount of exosomes lead to an enhancement level of chemoresistance-inducing factor—Snail—in epithelial cells. A new drug, GW4869, was found to reduce exosome biogenesis in gemcitabine-exposed CAFs and in other multiple cell lines [160]. Recently, a new method based on extracorporeal hemofiltration of exosomes from the circulation has been proposed, and this adaptive dialysis-like affinity platform technology requires further research [161]. Exosomes can be used as a system for the supply of anticancer agents and as a cell-free vaccine for cancer immunotherapy [162]. Currently, two clinical trials in Phase I and II (ClinicalTrials.gov Identifier: NCT01159288, ClinicalTrials.gov Identifier: NCT03608631) are being performed, where artificial exosomes against lung and pancreases cancers are used [163]. In order to express optional cargoes in exosomes, two protocols are used. The first of them is based on direct adding of the therapeutic agents, such as hydrophilic or hydrophobic drugs, and also siRNAs to exosome suspensions. For loading exosomes, many techniques have been utilized; for instance, incubation, sonication, electroporation, or a freeze/thaw cycle. The second, called an “indirect engineering method”, is focused on modifying parental cells, for example, via genetic manipulation to produce artificial exosomes [164]. For instance, Tian et al. purified exosomes from mouse immature dendritic cells and loaded them with doxycycline (Dox) via electroporation. Dox encapsulated into exosomes express peptide lysosome-associated membrane glycoprotein 2b on their surface, which allows for successful binding to αv integrin-positive breast cancer cells. Exosomes injected intravenously and suppling chemotherapeutic drugs to tumor tissues have higher anticancer effects and are deprived of overt toxicity in comparison to free Dox [165]. In addition, Shtam et al. demonstrated that siRNAs can be effectively delivered to target cells via exosomes and lead to selective genes silencing. However, the method of exosome loading needs to be further optimized [166].

### 3.5. Cytokines, Chemokines and Growth Factors

Due to the multitude of roles in the progression of cancers, TGFβ is considered an attractive target for developing cancer therapeutics. Until today, diverse strategies have been tested, among them, TGFβ inhibition at the translational level, sequestering the ligand with monoclonal antibodies, the use of inhibitors of TGFβ receptor kinases as well as monoclonal antibodies to inhibit TGFβ binding to the type II receptor. Galunisertib (LY2157299 monohydrate) is a potent and selective TGFβRI kinase inhibitor and is currently being tested in clinical trials (ClinicalTrials.gov Identifier: NCT02423343). An in vivo study conducted on breast, lung, and colon cancers and hepatocellular carcinoma confirmed its antitumor activity. In the first stage of the clinical trial, LY2157299 revealed promising antitumor activity in patients with glioma and had an acceptable safety profile [167,168,169]. The use of galunisertib in monotherapy and in combination with conventional chemotherapeutics are being tested.

Given that chemokines and their receptors are involved in several aspects of cancer biology, CCR4 blockade with monoclonal antibody (mogamulizumab) and inhibitor of chemokine receptor CXCR4 (AMD3100) are being tested in clinical studies. Through antibody-dependent cellular cytotoxicity, mogamulizumab destroys tumor cells and is currently used for the treatment of patients with refractory adult T-cell leukemia in Japan [170]. The anti-CCR4 antibody Affi-5 altered the phenotype of the myeloid cells towards antitumor and reduced the weight of cancer in renal carcinoma mice. These promising in vivo reports are the basis for a clinical trial of anti-CCR4 therapy in renal and other CCR4-overexpressing cancers [171]. Similarly, the safety and toxicity of the combination of the CXCR2 antagonist (AZD5069) with the androgen receptor antagonist enzalutamide is actually being investigated in patients with metastatic castration-resistant prostate (ClinicalTrials.gov Identifier: NCT03177187). In addition, AZD5069 is also being tested in patients with metastatic pancreatic ductal adenocarcinoma (ClinicalTrials.gov Identifier: NCT02583477). The most encouraging results of research under chemokine receptor inhibitors in the treatment of solid tumors are given when they are used in combination with chemotherapy. Interestingly, the last date revealed atypical chemokine receptors (ACKRs) as a crucial regulatory component of the chemokine network. They are considered as a target for innovative immunotherapy [172].

Secreted cytokines are messengers in the cross-talk between cancer and adjacent cells. Il-2 is known as a promotor of tumor eradication, and attempts to use Il-2 in anticancer treatment have been underway for a long time. However, up to today, its application in humans is limited due to its short half-life and harmful side effects. Clinical use of L19IL2 and L19TNF, the recombinant fusion protein of recombinant monoclonal antibody (L19) with the human recombinant interleukin-2 (IL-2) or the human tumor necrosis factor-alpha in patients suffering from metastatic melanoma is ongoing (ClinicalTrials.gov Identifier: NCT02076633). IL-6-targeted therapeutic strategies are also being intensively investigated. Tocilizumab, an anti-human IL-6 receptor antibody, inhibits lymph node metastasis [173]. The pharmacodynamic activity of this antibody, in combination with ipilimumab and nivolumab, the approved drugs for melanoma treatment, is now being assessed (ClinicalTrials.gov Identifier: NCT03999749). STAT6 mediates activation of IL-4 inducible genes and its inhibition can lead to blocking IL-4 as well as IL-13 signaling. Leflunomide and vorinostat are the most well-known inhibitors of STAT6 phosphorylation. They have multiple modes of action like tyrosine kinases inhibition or histone deacetylase (HDAC) inhibitors, but until now, their use alone in anticancer therapy is limited [97]. However, vorinostat, in combination with gefitinib, showed synergistic cytotoxicity in lung adenocarcinoma and hepatocarcinoma with mutant KRAS [174]. Similarly, anakinra, an IL-1 blocker, is applied as a treatment in many diseases such as rheumatoid arthritis, infections, and type 2 diabetes. The use of this agent in cancer treatment is still restricted, and up to today, there has been only one pilot study cited in clinicaltrials.gov for the safety of anakinra plus standard chemotherapy (ClinicalTrials.gov Identifier: NCT02021422).

Another target of anticancer therapy is VEGF that inhibitors effectively reduce blood vessel formation around the tumor. Currently, small-molecule inhibitors and monoclonal antibodies belong to this group of drugs. Sunitinib is a small-molecule inhibitor approved by FDA for use in gastrointestinal stromal tumors, advanced kidney cancer, and pancreatic neuroendocrine tumors [175], while Vandetanib with similar properties, for use against metastatic medullary thyroid cancer [176]. A typical representative of the second category of drugs targeting VEGF is a monoclonal antibody called bevacizumab that was first approved in the EU in 2005 and is now being used against colon, metastatic breast, and cervical cancers [177]. Available medicines are associated with the rare, but serious toxicities significantly linked with hypertension [178]. Despite many available drugs, creating a novel anti-VEGF treatment is needed, especially one that would be safe for longer-term adjuvant or maintenance treatment. Ramucirumab, a monoclonal antibody inhibiting VEGFR-2, is the first biological treatment approved in 2014 that exerts moderate survival benefits after first-line chemotherapy in gastroesophageal adenocarcinoma [179].

### 3.6. Metabolites-Mediated Communication

In cancers, enzymes of TCA are deregulated; thus they are a target of new therapeutic avenues. CPI-613 is a new compound with a structure similar to lipoic acid and the ability to inhibit pyruvate and α-ketoglutarate dehydrogenases. Now, CPI-613 is being investigated in Phase I and II clinical trials (ClinicalTrials.gov Identifier: NCT02168140, ClinicalTrials.gov Identifier: NCT01902381, ClinicalTrials.gov Identifier: NCT02232152, ClinicalTrials.gov Identifier: NCT01766219; ClinicalTrials.gov Identifier: Identifier: NCT01931787). Data from these trials emphasize that CPI-613 effectively shuts off mitochondria and deprives hematological cancer cells of energy [180], but its efficiency has not been confirmed in patients suffering from small-cell lung carcinoma [181]. Glutamine is a fuel source for the TCA cycle in many tumors and CB-839, an inhibitor of glutaminase, is used to block the conversion of glutamine to glutamate. This compound is under investigation in patients with hematological tumors (ClinicalTrials.gov Identifier: NCT02071927 and ClinicalTrials.gov Identifier: NCT02071888).

Lipids are also energy-rich molecules that are essential for cancer development. Previous research has revealed that sterol regulatory element-binding proteins (SREBPs) are upregulated in cancers, and inhibition of their activation led to the induction of cancer cell death and suppression of tumor growth. A promising approach is blocking the translocation of SREBP to the Golgi. Among SREBP activity inhibitors, betulin, fatostatin, and PF-429242, have promising anti-tumor effects in many preclinical studies [182,183]. Fatostatin was shown to inhibit cancer cell invasion and migration and to arrest prostate cancer cells at the G2/M checkpoint. In addition, it restrained the growth of pancreatic cancer [184]. However, so far, no clinical trials on SREBPs inhibitors in the treatment of human cancers have been published [185]. However, several compounds that attenuate the growth of malignant via inhibition of fatty acid synthase (FASN) have been tested. Unfortunately, most preclinical studies have failed; they revealed the pharmacological limitations of these compounds. More recently, a study on the next-generation inhibitors of FASN, TVB-3166, and TVB-2640, has shown their antitumor efficacy in models of breast and colorectal cancer [186]. Moreover, TVB-2640 is characterized by excellent tolerability and limited systemic toxicity in patients with advanced-stage solid malignant tumors (ClinicalTrials.gov Identifier: NCT02223247) [187].

In cancers, the upregulation of FASN is consistent with excess metabolism of arachidonic acid (AA) that can be converted to prostaglandins by the COX-dependent pathway. In oncology, the use of COX inhibitors to reduced PGE2 production has been hampered due to the risk of adverse side effects. However, in a preclinical study, specific pharmacological inhibition of microsomal prostaglandin E synthase-1 (mPGES-1) by non-toxic single drug CIII effectively blocked CAF-derived PGE2 production and modulated the neuroblastoma microenvironment towards being less tumor-promoting. Therefore, currently, mPGES-1 is considered a promising novel target for neuroblastoma therapy [188]. Despite intensive research on sphingolipid-signaling, there are only a few inhibitors of the sphingosine kinase (SK). Preclinical studies have indicated a potential application of one of them, called ABC294640, in multiple tumors, including prostate, colorectal, lung, ovarian, and liver cancers, and leukemia. Therefore, it is now under investigation in patients with advanced multiple myeloma (ClinicalTrials.gov Identifier: NCT02757326). Data have indicated that ABC294640 is well tolerated and achieves biologically relevant concentrations in human plasma. A Phase I trial revealed the chemotherapeutic efficacy of SK2 inhibition by ABC294640. ABC294640 increased the ceramide level that induces apoptosis in tumor cells and suppresses signaling through pERK and pAKT [189].

## 4. Concluding Remarks

**Figure 3:**
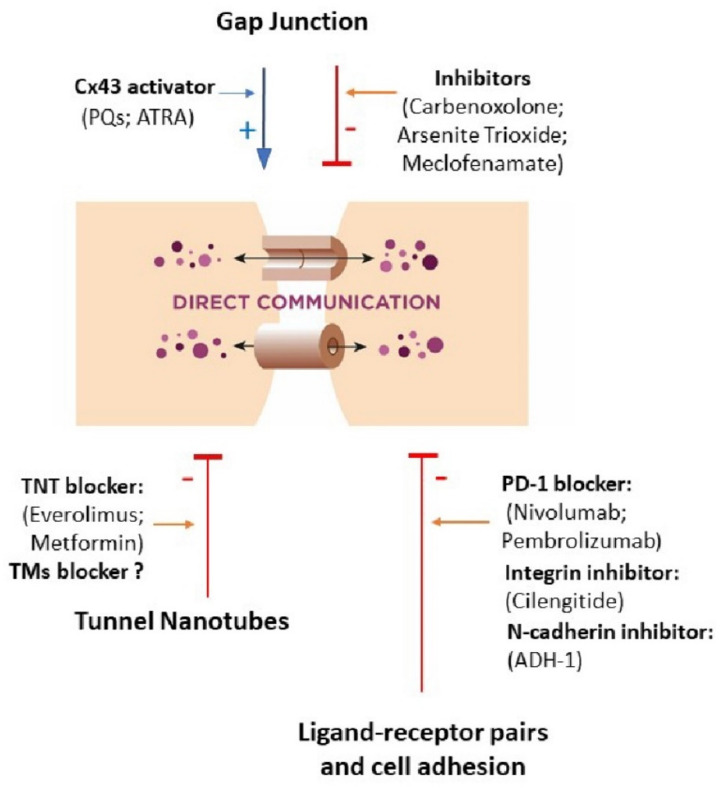
New therapeutic actions targeted at direct communication in the cancer microenvironment. Two ways of therapeutic options are being considered: the first is focused on attenuation or inhibition of cell–cell interaction (red lines), and the second is based on its intensification (blue arrows). ATRA—all-trans retinoic acid; Cx—connexin; TMs—tumor microtubes; TNT—tunnel nanotube.

**Figure 4:**
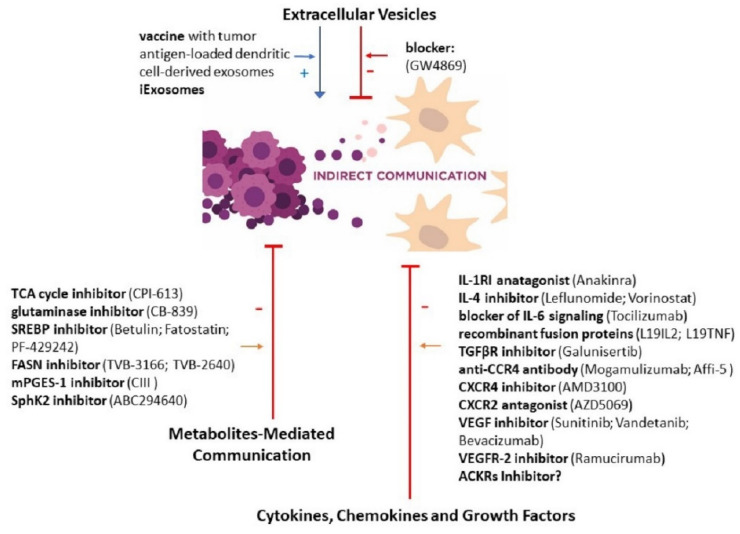
New therapeutic actions targeted at indirect communication in the cancer microenvironment. Two ways of therapeutic options are being considered: the first is focused on attenuation or inhibition of cell–cell interaction (red lines), and the second is based on its intensification for efficient delivery of the drug to tumor cells (blue arrows). ACKR—atypical chemokine receptor; iExosomes—exosomes derived from mesenchymal stromal cells with KrasG12D siRNA; FASN—fatty acid synthase; mPGES-1—microsomal prostaglandin E synthase-1; SphK2—sphingosine kinase 2; SREBP—sterol regulatory element-binding protein; TCA—tricarboxylic acid cycle.

The field of communication in the cancer microenvironment is a relatively new concept in tumor biology that is rapidly growing. Cancer–stroma cross-talk is an extremely complicated phenomenon, but different forms of cellular communication are highly expressed in cancer and clearly participate in cancerogenesis. The newest data do not support the old belief that cancerous cells are disconnected from neighboring cells and isolated from the environment. Today, we believe that cell–cell communication is critical to create the tumor niche [190]. Therefore, a novel medical approach (Figure 3 and Figure 4) focuses on the inhibition of cell–cell communication in cancer or the use of these routes of this communication as vectors of drug delivery to tumor cells. Special attention is paid to TNTs and exosomes to reduce cancer development and progression. Based on available data, we can recognize the benefits of therapeutic intervention in signaling molecules and organelles transfer and propose that medical therapy targeted intercellular communication should be a part of personalized oncology treatment. However, for further development of this concept, there is a need to develop reproducible experimental models that mimic human tumor and TME architecture, their heterogeneity, movement of biological fluids to study cell–cell cross-talks. TME differs between cancer types; therefore, cancer-specific models are needed to properly translate laboratory observations into in vivo situations. Research analyzing cell–cell interactions at single-cell level in tumor microenvironments and between cancer and TME cells should be further developed to recognize biologically relevant interactions associated with specific phenotypic outcomes. Some interactions cannot only be cancer-specific but also patient-specific, and their recognition would help to predict patient response to therapy. New therapeutic strategies should target both the cancer and its microenvironment; therefore, combined treatment algorithms should be a subject of research and nanomedicine seems to be promising as nanoparticles can deliver different drugs affecting different cells at the same time. However, other drug delivery systems made from synthetic and natural biomaterials should also be analyzed, and the effect of cancer microenvironment should be underlined.
